# Nutrient Composition and Quality Assessment of Royal Jelly Samples Relative to Feed Supplements

**DOI:** 10.3390/foods13121942

**Published:** 2024-06-20

**Authors:** Sampat Ghosh, Hyeonjeong Jang, Sukjun Sun, Chuleui Jung

**Affiliations:** 1 Agriculture Science and Technology Research Institute, Andong National University, Andong 36729, Gyeongsangbuk do, Republic of Korea; sampatghosh.bee@gmail.com; 2 Department of Plant Medicals, Andong National University, Andong 36729, Gyeongsangbuk do, Republic of Korea; jhj971008@naver.com (H.J.); scv6309@naver.com (S.S.)

**Keywords:** apiary, amino acid, api-products, minerals, royal jelly, stable carbon isotope (δ^13^C), quality, fructose, glucose, sucrose, 10-hydroxy 2-decenoic acid (10-HDA)

## Abstract

Royal jelly is a substance secreted by the hypopharyngeal and mandibular glands of nurse honey bees, serving as crucial nutritional source for young larvae, queen honey bees, and also valuable product for humans. In this study, the effect of the feed supplements on the nutritional composition and qualities of royal jelly was investigated. Two types of royal jelly samples were acquired: one from honey bees fed with sugar syrup as a feed supplement and the other from honey bees fed with honey. The production, harvesting, and storage of all royal jelly samples followed standard procedures. Parameters for quality assessment and nutritional value, including stable carbon isotopic ratio, moisture content, 10-hydroxy-2-decenoic acid (10-HDA) level, carbohydrate composition, amino acid composition, and mineral contents, were analyzed. The results revealed that despite variability in moisture content and carbohydrate composition, fructose was lower (2.6 and 4.1 g/100 g as is for sugar-fed and honey-fed royal jelly, respectively) and sucrose was higher (7.5 and 2.7 g/100 g as is for sugar-fed and honey-fed royal jelly, respectively) in the sugar-fed group. The stable isotope ratio (−16.4608‰ for sugar-fed and −21.9304‰ for honey-fed royal jelly) clearly distinguished the two groups. 10-HDA, amino acid composition, and total protein levels were not significantly different. Certain minerals, such as potassium, iron, magnesium, manganese, and phosphorus were higher in the honey-fed group. Hierarchical analysis based on moisture, sugar composition, 10-HDA, and stable carbon isotopes categorized the samples into two distinct groups. This study demonstrated that the feed source could affect the nutritional quality of royal jelly.

## 1. Introduction

Royal jelly (RJ), a substance secreted by the hypopharyngeal and mandibular glands of nurse honey bees (workers), serves as a crucial nutritional source for young larvae destined to become queen honey bees [1]. The process through which RJ induces larvae to become queens involves DNA methylation and altered gene expression, as demonstrated by an earlier study [2]. As nutrition plays a key role in the caste differentiation of honey bees, the chemical composition of RJ has attracted attention. Given the pivotal role of nutrition in honey bee caste differentiation, there is a growing interest in the chemical composition of RJ. 

The nutrient composition of RJ is of significant interest, particularly its protein content, mineral composition, and high fructose content. RJ also contains the principal fatty acid, 10-hydroxy-2-decenoic acid (10-HDA), known for its diverse biological activities such as antimicrobial properties and immune modulation [3,4,5]. These notable bio-functional properties, coupled with the growing awareness of natural products, have led to an increased demand for RJ and other hive products. China leads in RJ production, with other Asian countries notably increasing production over the past decade.

The exceptional biological properties of RJ have attracted considerable attention and commercial interest from various industries, including pharmaceuticals, functional foods, cosmetics, and manufacturing [6]. 

Studies suggest RJ has potential bio-functional benefits, including antioxidant properties, immune support, anti-inflammatory effects, and potential contributions to skin health, cognitive function, energy levels, and hormonal balance [7,8,9,10]. The primary functional role of royal jelly is frequently associated with the major royal jelly proteins (MRJPs). MRJPs exhibit diverse biological functions beyond their role in honey bee larval development. They, alongside royalactin, contribute significantly to royal jelly’s antibacterial activity, particularly against Gram-positive bacteria [11]. Notably, MRJP1 has been observed to promote cell proliferation and albumin production in rat hepatocytes, potentially serving as substitutes for growth factors in cell culture media [12]. Additionally, it exhibits antioxidant, anti-tumor, and immune-modulating properties [13]. MRJP2 and MRJP3 demonstrate immunomodulatory effects, with MRJP3 showing promise as an anti-allergic and anti-inflammatory agent [14,15]. While MRJP4 contributes nutritive components to royal jelly, its expression in the hypopharyngeal gland is relatively low compared to other MRJPs [16,17]. MRJP6, MRJP7, and MRJP8 appear to lack nutritional functions in *Apis cerana*, with MRJP8 and MRJP9 being considered ancestral members of the MRJP family [12].

This concern is reflected in the efforts of several countries, such as China, Switzerland, Bulgaria, Brazil, and Uruguay, to establish quality standards for RJ [6,18]. Leading producers of RJ include China, Japan, the USA, Canada, Australia, and certain European countries. While Korea’s RJ production is increasing, official statistics are not yet available.

Technological advancements, including the development of high-yielding honey bee genetic strains, modifications in feeding practices to increase RJ production, and improvements in harvesting techniques, have enhanced production [19,20,21]. However, it is equally important to maintain the quality and standards of RJ [22]. Nonetheless, there is currently no legislative framework for RJ standards in Korea. Consequently, it is essential to evaluate the chemical composition of RJ produced by local apiaries to assess its quality according to international standards and to investigate the factors influencing royal jelly quality. 

Furthermore, the nutritional composition of RJ, a protein-rich substance typically comprising 9–18% protein for fresh RJ [6], may be influenced by the honey bees’ dietary intake, particularly in terms of the protein content of their feed. Stimulating royal jelly production often involves providing selected colonies with supplementary feed, typically consisting of sugar syrup or honey, in addition to pollen patties. This practice ensures that the nurse bees receive adequate nourishment to produce high-quality royal jelly. Prior research by Ghosh and Jung [23] has demonstrated that the composition of pollen patties, derived from various bee pollen sources, impacts the peptide levels within RJ. Nevertheless, the impact on additional nutritional factors such as proximate composition, amino acid profile, 10-HDA, and minerals was not discernible, possibly because of the relatively consistent protein content between oak and rapeseed bee pollen, and consequently, the resulting pollen patties [23]. However, the impact of using honey and sugar syrup as feeding supplements on royal jelly quality remained to be determined. Honey, being nutrient-dense with compounds beyond carbohydrates compared to sugar syrup, could potentially enhance the quality and nutritional value of royal jelly. Therefore, to investigate this further, we collected RJ samples from 11 different commercial apiaries across diverse regions of South Korea to evaluate their nutritional characteristics. Stable carbon isotope analysis allowed us to categorize the samples into two groups, corroborated by information obtained from the respective apiaries regarding the diet of the honey bees. We identified samples produced by honey bees that were fed either honey or a sugar solution. Subsequently, we conducted a comparative analysis of the nutritional profiles of RJ from both feeding groups to ascertain whether the diet of honey bees influences the nutrient composition of RJ. 

## 2. Materials and Methods

### 2.1. Sample Collection

To conduct this study, eleven commercial apiaries were randomly selected from various locations across the country to ensure a diverse sample set. These apiaries were chosen based on their adherence to standard procedure for harvesting and storing royal jelly. Additionally, all the beekeepers involved were trained professionals running commercial operations. At least three samples (each weighing about 100 g) were collected from each apiary. 

RJ production in the apiaries adhered to standard protocols [24]. This procedure outlines the steps involved in producing royal jelly, from colony selection to storage, ensuring the highest-quality product for consumers: (a) Selection of colonies: The production of royal jelly (RJ) starts with the careful selection of robust and productive bee (*Apis mellifera ligustica*) colonies. These colonies need to have an ample number of nurse bees, which are essential for the production of RJ. (b) Supplementary feeding: To stimulate the production of royal jelly, the selected colonies are provided with supplementary feed. This feed usually includes sugar syrup or honey, along with pollen patties. This ensures that the nurse bees have sufficient nourishment to produce high-quality RJ. (c) Preparation for grafting: The procedure involves transferring young larvae from conventional honeycomb cells to specialized royal jelly cups within the hive. This transfer method is known as grafting. (d) Grafting: During grafting, beekeepers carefully move the young larvae into the royal jelly cups. This process is crucial for ensuring that the larvae receive the royal jelly necessary for their development into queen bees. (e) Harvesting: After a specific timeframe, typically spanning 24 to 72 h, the royal-jelly-filled cups are meticulously harvested from the hive. In this study, RJ samples were harvested at 72 h. This process requires precision to ensure the quality and purity of the royal jelly. (f) Storage: Once harvested, the royal-jelly-filled cups are stored at −20 °C to maintain their freshness and quality until further use or distribution. 

We obtained the samples of RJ in freezing boxes from commercial apiaries from different parts of the country. We conducted stable carbon isotope analysis according to the standardized method outlined later. This analysis enabled us to classify the samples into two groups, supported by information on the feed supplement from the respective apiaries about the diet of the honey bees. The RJ sampling address, geographical locations, environmental conditions at the time the RJ was harvested (June 2019), stable radioisotope ratio, and feed supplement information are given in Table 1. The sample numbers were reassigned based on the feeding information. We kept the samples at −20 °C until further processing. All glassware used for the chemical analyses was meticulously cleaned, and the chemicals used for analytical purposes were of pure HPLC grade. 

### 2.2. Moisture Content and Carbohydrate Composition

All the chemical analyses including moisture content (AOAC 934.01) and sugar analysis were conducted following the standard method by AOAC [25] and Codex [26], respectively. 

### 2.3. Radioactive Carbon Estimation by IRMS

We used Delta V Plus with a characterized ion source of 3 KV and monitored masses *m*/*z* 1~8 (Thermo Fisher Scientific, Waltham, MA, USA) with a continuous-flow isotope ratio mass spectrometer in order to determine δ^13^C (‰) in the honey samples. The isotopic ratio was measured following the standard method of the AOAC methodology (AOAC 998.12) [25]. Each honey sample was put in a ceramic boat and placed in the combustion system, where it was subjected to 600 mm of mercury (Hg) and oxygen over purified CuO at 700 °C, followed by a liquid nitrogen trap. The sample was heated to ≤850 °C, and CO_2_ was condensed in the liquid nitrogen trap. Further, the condensed CO_2_ underwent purification and was identified with a mass spectrometer specially designed for isotope ratio measurement. The mass spectrometer separates ionic forms of molecules according to their specific mass-to-charge ratio (*m*/*z*).

### 2.4. Amino Acid Analysis

The amino acid composition was estimated using a Sykam Amino Acid analyzer S433 (Sykam GmbH, Eresing, Germany) equipped with a Sykam LCA L-07 column following the standard method (AOAC 994.12) [25]. The samples (20 mg) were hydrolyzed in 6 N HCl for 24 h at 110 °C under a nitrogen atmosphere and then concentrated in a rotary evaporator. The concentrated samples were reconstituted with sample dilution buffer supplied by the manufacturer (physiological buffer 0.12 N citrate buffer, pH 2.20). The hydrolyzed samples were analyzed for amino acid composition. 

### 2.5. 10-Hydroxy-2-Decenoic Acid (10-HDA) Analysis

The determination of trans-10-hydroxy-2-decenoic acid content of RJ samples followed the established procedure by Zhou et al. [27].

### 2.6. Mineral Analysis

Minerals were analyzed following standard procedures according to the Korean Food Standard Codex [28]. Samples were digested with nitric and hydrochloric acid (1:3) at 200 °C for 30 min. Each sample was then filtered using Whatman filter paper (0.45 microns) and stored in washed glass vials before analyses could commence. The mineral contents were analyzed using an inductively coupled plasma-optical emission spectrophotometer (ICP-OES 720 series; Agilent; Santa Clara, CA, USA).

### 2.7. Statistical Analysis

Each experiment was conducted in triplicate to ensure the reliability of the results, and the data were presented as mean ± standard deviation. Statistical analysis to identify significant differences was performed using one-way ANOVA followed by post hoc testing. To ascertain the statistical significance between the two groups, a *t*-test assuming unequal variance was conducted. The null hypothesis was rejected when *p* ≤ 0.05. Hierarchical cluster analysis was employed to classify the RJ samples based on moisture content, sugar composition, 10-HDA content, and stable carbon isotope analysis. Statistical analyses were conducted using SPSS 16.0 software (IBM).

## 3. Results

### 3.1. Moisture Content

The moisture content of RJ samples varied across different samples, as illustrated in Table 2. Samples S1, S2, S3, H1, and H3 had moisture content values ranging from 57.3% to 59.4%, with slight differences among them. Samples S4, S5, H2, H4, H5, and H6 exhibited higher moisture content, ranging from 60.7% to 62.0%, with sample 9 showing the highest moisture content at 62.0%. Statistical analysis revealed significant differences in moisture content among the farms (df = 36, F = 5.389, *p* = 0.000). Sample S1 had the lowest moisture content, while sample H5 had the highest, indicating variability in the water content of RJ samples obtained from different sources. 

**Figure 1:**
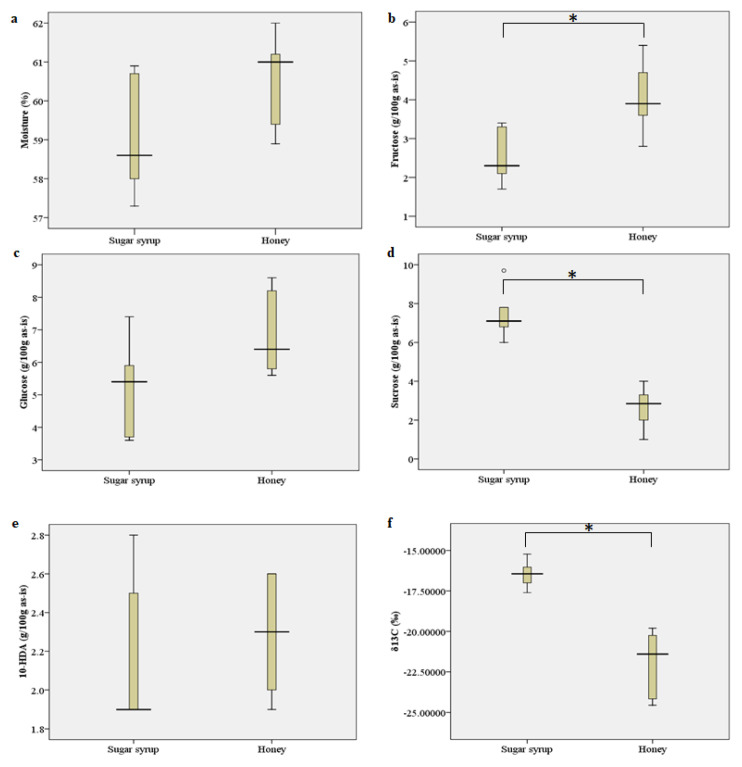
Physicochemical and chemical contents of royal jelly samples from two feeding treatments: sugar-syrup-fed honey bees and honey-fed honey bees. The parameters measured include (**a**) moisture, (**b**) fructose, (**c**) glucose, (**d**) sucrose, (**e**) 10-HDA, and (**f**) δ^13^C. * indicates significant difference.

While considering the treatment point of view, there was no significant difference in moisture content observed between the two sets of RJ samples (*p* = 0.132): those sourced from colonies fed sugar syrup and those from colonies fed honey as a supplement (Table 2 and Figure 1a).

### 3.2. Carbohydrate Content

The carbohydrate contents, fructose, glucose, and sucrose levels in RJ samples from different commercial farms are summarized in Table 3. Fructose and glucose were abundant in the case of six samples (H1–H6), while sucrose predominated in the other five (S1–S5) samples. Fructose content ranged from 1.7 to 5.4 g per 100 g of RJ on an as-is basis. Glucose content accounted for 3.6 to 8.6 g per 100 g of RJ. The total carbohydrate content, including these three compounds, was within the range of 12.4 to 20.4%. These results demonstrate considerable variability in fructose content among the RJ samples obtained from different samples (df = 36, F = 12.076, *p* = 0.000). Samples H1 and H6 exhibited relatively higher fructose levels compared to the others, while sample S4 showed the lowest fructose content. The standard deviations indicate the degree of variability within each sample. These results illustrate variations in glucose content among the RJ samples obtained from different samples (df = 36, F = 3.988, *p* = 0.002). Samples H1 and H6 displayed relatively higher glucose levels compared to the others, while samples S3 and S4 exhibited the lowest glucose content. Similarly, these results demonstrate variability in sucrose content among the RJ samples obtained from different samples (df = 36, F = 18.970, *p* = 0.000). Samples S1–S5 displayed relatively higher sucrose levels, while samples H1 and H6 exhibited the lowest sucrose content. The standard deviations indicate the degree of variability within each sample. 

A significant difference in fructose and sucrose content of RJ samples was found between the two groups: those sourced from colonies fed sugar syrup and those from colonies fed honey as a supplement (Table 3 and Figure 1b,d). Sucrose content was found to be statistically higher in RJ obtained from colonies fed with sugar syrup (7.5 g/100 g of RJ on an as-is basis) than in that obtained from colonies fed with honey (2.7 g/100 g of RJ on an as-is basis). Conversely, the reverse was observed for fructose. On the other hand, glucose and total sugar content were found to be consistent in both groups of RJ samples (Table 3 and Figure 1c).

### 3.3. 10-Hydroxy 2-Decenoic Acid

10-HDA, the predominant fatty acid, constitutes one of the significant components of RJ. Analysis presented in Table 4 delineated the 10-HDA content of RJ samples procured from commercial apiaries in South Korea. The concentration of 10-HDA ranged from 1.9 to 2.6 g per 100 g of RJ on an as-is basis (df = 36, F = 7.487, *p* = 0.000). Notably, 10-HDA exceeds 2 g per 100 g of RJ in seven samples, while in others, it remained at 1.9 g per 100 g. When examining the treatment aspect, no significant difference in 10-HDA content was observed between both groups of RJ samples (Table 4 and Figure 1e).

### 3.4. Radioactive Carbon Estimation

The radiocarbon value, represented by the stable radioisotope ^13^C in per mil. (per thousand) (‰), ranged from −15.2247 to −24.5570, as detailed in Table 4. Generally, there was a discernible correlation (0.902) observed between the sucrose content and the ^13^C value across the RJ samples. Specifically, samples with lower sucrose content tended to exhibit lower values for ^13^C (‰). These values indicate variations in the stable isotopic composition of carbon among the royal jelly samples sourced from different samples (df = 35, F = 45.794, *p* = 0.000). The standard deviations provided offer insights into the precision of the measurements. The stable carbon isotope (^13^C δ‰) value was significantly lower in RJ samples sourced from colonies fed with honey (−21.9304) compared to those obtained from colonies fed with sugar syrup (−16.4608) (Table 4 and Figure 1f).

**Figure 2:**
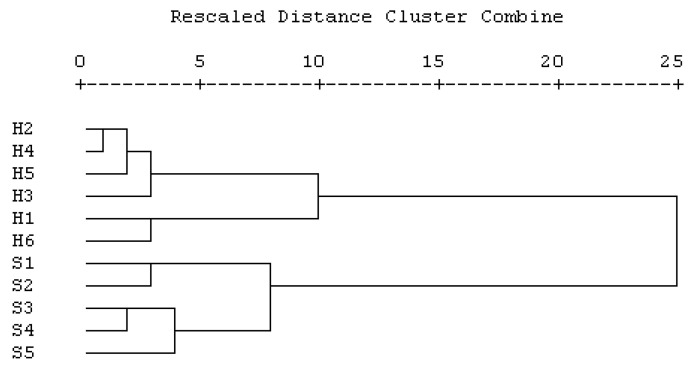
Hierarchical analysis of royal jelly samples based on moisture, sugar composition (fructose, glucose, and sucrose), 10-HDA, and stable carbon isotope analysis.

Incorporating factors such as moisture content, sugar composition (fructose, glucose, and sucrose), 10-HDA, and stable carbon isotopes revealed intriguing patterns. Hierarchical analysis delineated two distinct categories among the samples under study, as illustrated in Figure 2. Samples S1–S5 formed one cohesive group, characterized by a prevalence of sucrose among the sugars and higher stable carbon isotopic values. In contrast, samples H1–H6 constituted the other category, exhibiting a divergent profile in terms of sugar composition and stable carbon isotopes.

### 3.5. Amino Acid Composition

Altogether, seventeen amino acids were estimated, as illustrated in Table 5. Tryptophan was not estimated, and methionine and cysteine were not determined entirely, presumably because of the acid hydrolysis process. RJ samples S3 and H3 exhibited elevated levels of lysine compared to other essential amino acids, whereas in other samples, leucine was the most prevalent essential amino acid, followed by lysine. As for all amino acids, aspartic acid was identified as the most predominant, followed by glutamic acid. No significant differences were observed for individual amino acids as well as the total amino acid content in RJ samples obtained from the two groups (Table 5).

### 3.6. Minerals Content

Our analysis delved into the mineral composition of RJ, yielding insights that paint a nuanced picture of its nutritive profile. Table 6 encapsulates our findings, revealing intriguing variations in mineral content across the tested RJ samples, with zinc being the exception. The mineral levels, including calcium, copper, iron, potassium, magnesium, manganese, sodium, and phosphorus, except zinc, exhibited noteworthy diversity among the samples (calcium: df = 36, F = 6.116, *p* = 0.000; magnesium: df = 36, F = 9.606, *p* = 0.000; potassium: df = 35, F = 14.476, *p* = 0.000; sodium: df = 36, F = 12.139, *p* = 0.000; phosphorus: df = 36, F = 8.779, *p* = 0.000; iron: df = 36, F = 2.931, *p* = 0.013; zinc: df = 36, F = 1.630, *p* = 0.153; copper: df = 36, F = 5.933, *p* = 0.000; manganese: df = 36, F = 3.844, *p* = 0.003). Calcium, an essential mineral for bone health, ranged from 11.5 to 15.5 mg per 100 g of RJ, while sodium content remained relatively low at 1.7–2.6 mg per 100 g. In contrast, potassium emerged as a standout, boasting levels ranging from 261.9 to 362.0 mg per 100 g, indicative of its abundance in RJ samples. This high potassium content, coupled with low sodium levels, suggests a potentially favorable K/Na ratio for human health.

Moreover, phosphorus, crucial for bone and tooth health, showcased a significant presence, ranging from 204.9 to 280.1 mg per 100 g, with marginal significance. Magnesium, another vital mineral contributing to various physiological functions, demonstrated levels ranging from 27 to 38.4 mg per 100 g.

In the realm of micro-minerals, zinc prevailed, accounting for 2.3 to 2.7 mg per 100 g of RJ, while iron content fell within the range of 0.9 to 1.2 mg per 100 g. Copper and manganese were also discernible in the samples, albeit to a lesser extent.

RJ samples from honey bees fed with honey contained a significantly higher amount of potassium compared to those from bees fed with sugar syrup. Additionally, the levels of iron, manganese, and magnesium were marginally higher in the RJ produced by honey-fed honey bees compared to that of sugar-syrup-fed bees (Table 6).

## 4. Discussion

RJ samples derived from colonies fed with sugar syrup showed significantly elevated levels of sucrose and stable carbon radioisotope values and lower levels of fructose compared to samples from colonies fed with honey as a feed supplement. On the other hand, royal jelly samples produced by honey bees fed with honey, characterized by higher fructose, lower sucrose, and elevated levels of certain minerals, indicate that nutrient-dense honey might positively impact the quality and nutritional value of royal jelly. 

Moisture content was found to be in agreement with previously published scientific reports on RJ [29,30]. However, the value obtained for RJ samples in the present study was less than the moisture content reported in some other studies and standards [22,31,32]. Overall, the moisture content of the fresh RJ is within the range of 60 to 70% [6]. The slightly lower moisture content of our royal jelly can be attributed to the fact that it was harvested from temperate regions with relatively low relative humidity (RH). This likely explains why the moisture content of royal jelly samples in this study falls on the lower end of the range reported in the scientific literature. For instance, honey harvested from tropical regions with high RH and temperatures requires honey bees to expend more energy to reduce moisture during ripening compared to honey from temperate regions with lower RH and temperatures. This indicates that indigenous honey bees in tropical areas might ultimately cap honey at higher moisture levels than those traditionally shown for *A. mellifera* honey [33]. Moisture content is important to maintain the freshness and consistency of RJ. Higher moisture content may lead to faster spoilage. The moisture content of the RJ samples in this study was in the lower margin, which implies that it could potentially retain beneficial properties for human consumption or other applications, and at the same time, it helps increase preservation.

Sabatini et al. [6] reported that total fructose, glucose, and sucrose content of RJ accounted for 7 to 18%. Based on the available literature, Sabatini et al. [6] also provided a range for fructose (3–13%), glucose (4–8%), and sucrose (0.5–2%) content of RJ. Wytrychowski et al. [34] reported the carbohydrate content in commercial RJ as follows: fructose ranged from 2.41% to 7.53%, averaging 5.42%; glucose ranged from 3.22% to 7.62%, averaging 5.70%; and sucrose ranged from not detected (n.d.) to 3.85%, averaging 1.42%. For Italian RJ, fructose ranged from 2.73% to 5.10%, averaging 4.04%; glucose ranged from 2.00% to 5.85%, averaging 4.55%; and sucrose ranged from 0.07% to 3.18%, averaging 0.93%. In French RJ, fructose ranged from 2.80% to 6.04%, averaging 4.55%; glucose ranged from 4.17% to 7.40%, averaging 5.79%; and sucrose ranged from n.d. to 1.29%, averaging 0.16% [34]. In this study, however, a contrasting scenario with respect to sucrose content was revealed in case of six samples where significantly higher sucrose content was found in RJ. The higher sucrose content presumably depends on the sugar solution feeding of honey bee. The consumption of sugar syrup as a feeding supplement for honey bees results in royal jelly with significantly higher sucrose and lower fructose content compared to the honey-fed group. This suggests that the high sucrose content in the feed is likely incorporated into the synthesis of royal jelly. For instance, sugar-fed honey bees produce honey with notably high sucrose levels and low fructose levels [35], which aligns with the findings of this study on royal jelly. However, the specific metabolic pathways involved in this process have yet to be investigated. The observation of higher sucrose levels in RJ from sugar-syrup-fed bees aligns with the findings of Daniele and Casabianca [36], who demonstrated elevated sucrose content in RJ from the hive fed with cane sugar. Typically, RJ is considered to contain a little higher amounts of fructose than glucose [30], a trend consistently observed in our routine laboratory analyses (CJ, unpublished data), but this study revealed a higher glucose content relative to fructose, a pattern also noted in several earlier studies despite variations in the fructose-to-glucose ratio [34,36,37,38,39,40]. Based on the carbohydrate composition obtained in the present study, we assume that it is a common practice to feed honey bees with sugar solution, which in turn is being reflected in the sugar composition of RJ.

Sabatini et al. [5] reported that for fresh RJ, 10-HDA content should be more than 1.4%. Wang et al. [30] estimated 10-HDA for 2-day-old RJ on a fresh-weight basis 3.72 g per 100 g, but after that, it was within the range of 2.10 to 2.58%. The difference in 10-HAD content might be due to geographical location, honey bee subspecies, and flora, which significantly influence the 10-HAD content of royal jelly, as demonstrated by Wei et al. [41]. 10-HDA also exhibits functional properties in regard to human health like bacteriocide and anti-inflammatory activity in human colon cancer cells [2], anti-inflammatory effect [42], melanogenesis inhibitor [8], and antiproliferative effect on human neuroblastoma cells [43].

Stable carbon isotope analysis detects honey adulteration by measuring the ratio of ^13^C to ^12^C isotopes. Natural honey from C3 plants has a distinct isotopic signature, while common adulterants like corn syrup or cane sugar (from C4 plants) have a different isotopic ratio. By comparing the isotopic ratios, deviations from the expected values can indicate the presence of adulterants [44]. We hypothesize that if honey bees are fed with a sugar supplement, the royal jelly they produce will have a high ^13^C content (low absolute value). Conversely, if they are fed with a honey supplement, the royal jelly will have a low ^13^C content (high absolute value), similar to what is observed in honey [44]. In our current investigation, we found a significant difference in ^13^C stable isotope levels between the two types of royal jelly.

Not many reports are available on the amino acid composition of RJ. Sabatini et al. [5] reported that the total protein content of fresh RJ was within the range of 9–18%. Given the potential for overestimation when using different methods to measure total protein content, since nitrogen may be present in non-protein compounds also [45], assessing the total amino acid content, particularly essential and non-essential proteinogenic amino acids, offers a more accurate alternative for understanding nutritional value. The total amino acid content for the RJ samples was within the range of 10.7 to 13 g per 100 g of RJ on an as-is basis, which is in agreement to the protein content (11.6–12.2%) of harvested RJ reported by Howe et al. [29]. The results of this study align with the recent findings of Wang et al. [46], which demonstrated no significant difference in the majority of amino acids in RJ between the sucrose-fed and honey-fed treatment groups. Overall, essential amino acids accounted for 40.7 to 43.3% of the total amino acids. Aspartic acid was found to be predominant, followed by glutamic acid. Except tryptophan, all the essential amino acids were present in the RJ samples. Among the essential amino acids, leucine was the most abundant. A similar distribution pattern of amino acids was found in previous studies [32,47]. Essential amino acids are indispensable for human health, serving as the fundamental building blocks of proteins and participating in numerous physiological functions. Lysine, for instance, is crucial for collagen synthesis, tissue repair, and calcium absorption, impacting bone health and wound healing, supporting immune function, among others [48,49]. Branched-chain amino acids (BCAAs) like leucine, valine, and isoleucine are vital for muscle protein synthesis, energy production, and exercise performance [50]. Additionally, amino acids contribute to neurotransmitter production, immune function, and hormone regulation [51]. Since the body cannot synthesize essential amino acids endogenously, their intake through dietary sources is imperative for maintaining overall health and wellbeing, supporting growth, development, and optimal bodily function. Therefore, consumption of RJ could be beneficial for human health.

The findings of this study indicated that there was no impact of feeding on 10-HDA and amino acid levels, suggesting that the metabolic pathways related to lipid and protein synthesis remain largely unaffected by the type of feed provided. However, a comprehensive investigation into the specific metabolic pathways influenced by different feeding practices has yet to be conducted. This future research could provide deeper insights into how various feeds affect the overall metabolic processes in honey bees.

The mineral contents, which are nutritionally significant, of the royal jelly samples examined in this study were consistent with those reported in previous research [23]. The differences in mineral content, particularly potassium, iron, manganese, and magnesium, between the two groups may be attributed to the higher nutrient density, especially in minerals in honey compared to sugar syrup. RJ holds a pivotal role in human health owing to its rich mineral content. Essential minerals such as calcium, phosphorus, and magnesium support bone health by aiding in bone formation and density. Potassium is essential for maintaining fluid balance, nerve function, and muscle contraction, including heart rhythm. It also helps regulate blood pressure and supports bone health by neutralizing acids that can deplete calcium. RJ’s potassium-to-sodium ratio could help maintain electrolyte balance, crucial for nerve function and blood pressure regulation. Additionally, minerals like zinc, iron, and copper facilitate various metabolic processes, bolstering immunity and energy production. Moreover, RJ’s provision of antioxidants, including copper, manganese, and zinc, combats oxidative stress, promoting healthy aging [7,23]. These minerals also contribute to hormonal balance and overall wellbeing. In essence, integrating RJ into the diet offers a comprehensive array of minerals vital for optimal physiological functioning and resilience against illness. 

## 5. Conclusions

This comprehensive analysis sheds light on the diverse chemical and nutritional profiles of RJ samples in South Korea. Variability in moisture content, carbohydrate composition, 10-HDA levels, radiocarbon values, amino acid composition, and mineral content reflects the influence of geographical and environmental factors on RJ composition. The results indicate that the differences observed in the carbohydrate composition, particularly the lower fructose and higher sucrose levels in the sugar-fed group, along with the distinct stable isotope ratios, indicate that honey-fed royal jelly may have superior nutritional properties and overall quality. The hierarchical analysis revealed distinct categorization of samples based on key parameters, primarily in feed supplement, providing insights into the potential factors driving compositional differences in RJ production. Based on the findings of this study, we suggest that using honey as a feed supplement may enhance the quality of royal jelly compared to using sugar syrup. These findings underscore the importance of understanding regional variations in RJ composition for quality control and potential health benefits associated with its consumption. Further research exploring the underlying mechanisms contributing to these compositional differences is warranted to optimize RJ production and utilization. 

## Figures and Tables

**Table 1:** Detail of sampling address, geographical location, environmental conditions in June 2019, stable radioisotope ratio ^13^C (δ‰), and feed supplement of commercial royal jelly samples.

Sample	Sampling Address	Geographical Location		Temperature (°C)			Relative Humidity (%)		Stable Radioisotope Ratio (^13^C)	Feed	Reassigned Sample No.
		Longitude (°E)	Latitude (°N)	Max.	Min.	Avg.	Min.	Avg.			
1	Gyeonggi, Anseong	37.085	127.425	32.9	11	22	27	70	−16.4420 ± 0.061	Sugar	S1
2	Gyeongbuk, Sangju	36.465	128.093	33.4	12.5	22	27	73	−24.5570 ± 0.294	Honey	H1
3	JeonBuk, Namwon	35.384	127.585	32.5	11.2	21.7	19	73	−17.6085 ± 0.232	Sugar	S2
4	Daegu, Namgu	35.834	128.572	35.7	14.5	22.8	16	65	−20.7902 ± 0.671	Honey	H2
5	Gangwon, Yeongwol	37.214	128.333	32.5	9	20.9	19	69	−22.0152 ± 0.3836	Honey	H3
6	Gyeonggi, Pyeongtaek	36.935	126.958	33.1	12.1	21.9	24	70	−15.2247 ± 0.1136	Sugar	S3
7	Gyeonggi, Yeoju	37.364	127.591	31.4	10.4	21.1	27	70	−16.0297 ± 0.642	Sugar	S4
8	Gyeongbuk, Yeongcheon 1	35.895	129.007	35.5	11.8	21.9	16	65	−20.2528 ± 1.6891	Honey	H4
9	Gyeongbuk, Andong	36.530	128.750	32.7	10.5	21.5	21	67	−19.7995 ± 0.4675	Honey	H5
10	Gyeongbuk, Yeongcheon 2	35.894	129.009	35.5	11.8	21.9	16	65	−16.9992 ± 0.209	Sugar	S5
11	Gyeongbuk, Pohang	36.232	129.282	32.4	14.6	21.6	25	75	−24.1677 ± 0.355	Honey	H6

**Table 2:** Moisture content (% as-is basis) of royal jelly samples (superscripts indicates significant differences, *p* < 0.05).

Feed Supplement	Sample	Moisture Content
Sugar syrup	S1	57.3 ± 1.73 ^a^
	S2	58.0 ± 0.92 ^ab^
	S3	58.6 ± 1.08 ^ab^
	S4	60.9 ± 0.78 ^cde^
	S5	60.7 ± 1.08 ^cde^
Honey	H1	58.9 ± 0.17 ^abc^
	H2	61.0 ± 0.59 ^cde^
	H3	59.4 ± 1.10 ^bcd^
	H4	61.0 ± 1.49 ^de^
	H5	62.0 ± 1.48 ^e^
	H6	61.2 ± 0.98 ^de^
Mean (sugar syrup)		59.1 ± 1.62
Mean (honey)		60.6 ± 1.18
*p* *		0.132

* *p* value (two-tail) of the *t*-test, assuming unequal variance conducted between the two treatment groups.

**Table 3:** Sugar composition (g/100 g on an as-is basis) of royal jelly samples (superscripts indicates significant differences, *p* < 0.05; lower case superscript indicates the significant difference among the RJ samples and upper case superscript indicates significant difference between treatment groups).

Feed Supplement	Sample	Fructose	Glucose	Sucrose	Total	F:G
Sugar syrup	S1	3.3 ± 0.43 ^cd^	7.4 ± 1.53 ^bcd^	9.7 ± 1.37 ^e^	20.4	0.45
	S2	2.3 ± 1.00 ^ab^	5.9 ± 3.19 ^abc^	7.8 ± 0.30 ^d^	16.0	0.39
	S3	2.1 ± 0.02 ^ab^	3.7 ± 0.91 ^a^	6.8 ± 1.30 ^d^	12.6	0.57
	S4	1.7 ± 0.64 ^a^	3.6 ± 0.76 ^a^	7.1 ± 1.20 ^d^	12.4	0.47
	S5	3.4 ± 0.36 ^cd^	5.4 ± 0.42 ^ab^	6.0 ± 1.44 ^d^	14.8	0.63
Honey	H1	4.7 ± 0.64 ^ef^	8.2 ± 2.08 ^cd^	2.0 ± 0.35 ^ab^	14.9	0.57
	H2	3.6 ± 0.87 ^cd^	5.8 ± 0.60 ^abc^	3.0 ± 0.57 ^abc^	12.4	0.62
	H3	3.9 ± 0.46 ^de^	6.7 ± 1.62 ^bcd^	2.7 ± 1.13 ^ab^	13.3	0.58
	H4	3.9 ± 0.29 ^de^	5.6 ± 0.48 ^ab^	3.3 ± 1.63 ^bc^	12.8	0.70
	H5	2.8 ± 0.16 ^bc^	6.1 ± 0.57 ^abcd^	4.0 ± 0.20 ^c^	12.9	0.46
	H6	5.4 ± 0.78 ^f^	8.6 ± 1.28 ^d^	1.0 ± 0.50 ^a^	15.0	0.63
Mean (sugar syrup)		2.6 ± 0.75 ^B^	5.2 ± 1.60	7.5 ± 1.40 ^A^	14.8 ± 3.26	0.5 ± 0.10
Mean (honey)		4.1 ± 0.90 ^A^	6.8 ± 1.28	2.7 ± 1.05 ^B^	13.6 ± 1.12	0.6 ± 0.08
*p* *		0.015	0.102	0.000	0.319	0.130

* *p* value (two-tail) of the *t*-test, assuming unequal variance conducted between the two treatment groups.

**Table 4:** 10-Hydroxy-2-decenoic acid content (g/100 g on an as-is basis) and ^13^C (δ‰) of royal jelly samples (superscripts indicates significant differences, *p* < 0.05; lower case superscript indicates the significant difference among the RJ samples and upper case superscript indicates significant difference between treatment groups).

Feed Supplement	Sample	10-HDA	Stable Radioisotope (^13^C)
Sugar syrup	S1	1.9 ± 0.12 ^a^	−16.4420 ± 0.061 ^def^
	S2	1.9 ± 0.25 ^a^	−17.6085 ± 0.232 ^d^
	S3	2.5 ± 0.17 ^bc^	−15.2247 ± 0.1136 ^f^
	S4	2.8 ± 0.07 ^c^	−16.0297 ± 0.642 ^ef^
	S5	1.9 ± 0.10 ^a^	−16.9992 ± 0.209 ^de^
Honey	H1	2.6 ± 0.14 ^bc^	−24.5570 ± 0.294 ^a^
	H2	2.6 ± 0.29 ^bc^	−20.7902 ± 0.671 ^bc^
	H3	2.0 ± 0.35 ^a^	−22.0152 ± 0.3836 ^b^
	H4	2.4 ± 0.19 ^b^	−20.2528 ± 1.6891 ^c^
	H5	2.2 ± 0.27 ^ab^	−19.7995 ± 0.4675 ^c^
	H6	1.9 ± 0.09 ^a^	−24.1677 ± 0.355 ^a^
Mean (sugar syrup)		2.2 ± 0.42	−16.4608 ± 0.911 ^B^
Mean (honey)		2.3 ± 0.30	−21.9304 ± 2.028 ^A^
*p* *		0.723	0.001

* *p* value (two-tail) of the *t*-test, assuming unequal variance conducted between the two treatment groups.

**Table 5:** Amino acid composition (g/100 g on an as-is basis) of royal jelly samples.

Feed Supplement	Sugar Syrup						Honey							*p* **
Amino Acid	S1	S2	S3	S4	S5	Mean (Sugar Syrup)	H1	H2	H3	H4	H5	H6	Mean (Honey)	
Val *	0.6 ± 0.03	0.7 ± 0.02	0.7 ± 0.04	0.6 ± 0.09	0.7 ± 0.05	0.66 ± 0.05	0.6 ± 0.06	0.7 ± 0.02	0.7 ± 0.02	0.7 ± 0.06	0.8 ± 0.07	0.7 ± 0.04	0.70 ± 0.06	0.290
Thr *	0.6 ± 0.08	0.7 ± 0.05	0.6 ± 0.05	0.4 ± 0.14	0.5 ± 0.04	0.56 ± 0.11	0.5 ± 0.10	0.6 ± 0.13	0.7 ± 0.04	0.6 ± 0.10	0.7 ± 0.13	0.6 ± 0.07	0.62 ± 0.08	0.373
Leu *	0.9 ± 0.06	1.0 ± 0.02	1.0 ± 0.01	0.9 ± 0.09	1.0 ± 0.05	0.96 ± 0.05	1.0 ± 0.07	1.0 ± 0.05	1.0 ± 0.03	0.9 ± 0.08	1.1 ± 0.10	1.0 ± 0.05	1.00 ± 0.06	0.290
Ile *	0.6 ± 0.05	0.6 ± 0.01	0.6 ± 0.00	0.6 ± 0.31	0.6 ± 0.03	0.60 ± 0.00	0.6 ± 0.04	0.6 ± 0.04	0.6 ± 0.02	0.6 ± 0.04	0.7 ± 0.06	0.6 ± 0.04	0.62 ± 0.04	0.363
Lys *	0.8 ± 0.03	1.0 ± 0.01	1.3 ± 0.06	0.9 ± 0.18	1.0 ± 0.03	1.00 ± 0.19	0.9 ± 0.06	0.9 ± 0.07	1.1 ± 0.18	0.8 ± 0.05	1.0 ± 0.08	1.0 ± 0.07	0.95 ± 0.10	0.614
Phe *	0.6 ± 0.04	0.6 ± 0.04	0.5 ± 0.02	0.6 ± 0.17	0.6 ± 0.03	0.58 ± 0.04	0.6 ± 0.03	0.6 ± 0.03	0.6 ± 0.02	0.5 ± 0.05	0.6 ± 0.06	0.6 ± 0.03	0.58 ± 0.04	0.901
His *	0.3 ± 0.02	0.4 ± 0.14	0.3 ± 0.01	0.4 ± 0.49	0.3 ± 0.01	0.34 ± 0.05	0.3 ± 0.02	0.3 ± 0.02	0.3 ± 0.01	0.3 ± 0.02	0.3 ± 0.02	0.3 ± 0.01	0.30 ± 0.00	0.178
Met *	0.1 ± 0.04	0.1 ± 0.05	0.1 ± 0.03	0.1 ± 0.05	0.1 ± 0.03	0.10 ± 0.00	0.1 ± 0.02	0.1 ± 0.01	0.1 ± 0.03	0.1 ± 0.02	0.1 ± 0.04	0.1 ± 0.04	0.10 ± 0.00	0.076
Tyr	0.5 ± 0.04	0.5 ± 0.03	0.5 ± 0.01	0.5 ± 0.01	0.5 ± 0.04	0.50 ± 0.00	0.5 ± 0.04	0.5 ± 0.04	0.5 ± 0.03	0.4 ± 0.07	0.5 ± 0.07	0.5 ± 0.02	0.48 ± 0.04	0.363
Asp	1.6 ± 0.11	1.7 ± 0.10	1.6 ± 0.01	1.4 ± 0.16	1.7 ± 0.08	1.60 ± 0.12	1.6 ± 0.09	1.6 ± 0.07	1.7 ± 0.06	1.6 ± 0.12	1.9 ± 0.17	1.7 ± 0.26	1.68 ± 0.12	0.284
Ser	0.8 ± 0.04	0.8 ± 0.02	0.8 ± 0.03	0.7 ± 0.09	0.8 ± 0.18	0.78 ± 0.04	0.7 ± 0.11	0.8 ± 0.08	0.8 ± 0.03	0.8 ± 0.08	1.0 ± 0.16	0.8 ± 0.10	0.82 ± 0.10	0.441
Glu	1.2 ± 0.07	1.4 ± 0.03	1.3 ± 0.02	1.1 ± 0.10	1.3 ± 0.03	1.26 ± 0.11	1.2 ± 0.09	1.2 ± 0.07	1.3 ± 0.04	1.4 ± 0.11	1.6 ± 0.13	1.3 ± 0.06	1.33 ± 0.15	0.382
Pro	0.8 ± 0.08	0.6 ± 0.05	0.9 ± 0.03	0.7 ± 0.08	0.9 ± 0.08	0.78 ± 0.13	0.8 ± 0.12	0.5 ± 0.03	1.0 ± 0.04	0.7 ± 0.06	0.9 ± 0.10	0.9 ± 0.07	0.80 ± 0.18	0.835
Gly	0.4 ± 0.03	0.4 ± 0.06	0.4 ± 0.02	0.4 ± 0.04	0.4 ± 0.01	0.40 ± 0.00	0.4 ± 0.03	0.4 ± 0.02	0.4 ± 0.02	0.4 ± 0.04	0.5 ± 0.03	0.4 ± 0.02	0.42 ± 0.04	0.363
Ala	0.4 ± 0.03	0.4 ± 0.04	0.4 ± 0.03	0.3 ± 0.05	0.4 ± 0.01	0.38 ± 0.04	0.4 ± 0.03	0.4 ± 0.02	0.4 ± 0.03	0.4 ± 0.03	0.5 ± 0.04	0.4 ± 0.03	0.42 ± 0.04	0.197
Cys	0.1 ± 0.01	0.1 ± 0.06	0.1 ± 0.01	0.1 ± 0.08	0.2 ± 0.03	0.12 ± 0.04	0.1 ± 0.01	0.1 ± 0.03	0.1 ± 0.01	0.1 ± 0.05	0.2 ± 0.03	0.1 ± 0.03	0.12 ± 0.04	0.901
Arg	0.6 ± 0.04	0.9 ± 0.37	0.7 ± 0.01	0.8 ± 0.05	0.7 ± 0.02	0.74 ± 0.11	0.6 ± 0.03	0.7 ± 0.04	0.7 ± 0.02	0.6 ± 0.06	0.7 ± 0.06	0.7 ± 0.03	0.67 ± 0.05	0.241
Total	10.7	11.9	11.8	10.4	11.8	11.32 ± 0.71	11.0	11.1	12.0	11.0	13.0	11.6	11.62 ± 0.79	0.528

* indicates the essential amino acid for human; ** *p* value (two-tail) of the *t*-test, assuming unequal variance conducted between the two treatment groups; *p* value within the sugar-syrup-fed group: Asp = 0.000, Thr = 0.001, Ser = 0.047, Glu = 0.000, Pro = 0.000, Gly = 0.113, Ala = 0.011, Val = 0.000, Cys = 0.022, Met = 0.240, Ile = 0.829, Leu = 0.000, Tyr = 0.973, Phe = 0.931, His = 0.744, Lys = 0.058, and Arg = 0.689; *p* value within the honey-fed group: Asp = 0.012, Thr =0.010, Ser = 0.002, Glu = 0.000, Pro = 0.000, Gly = 0.012, Ala = 0.024, Val = 0.000, Cys = 0.000, Met = 0.024, Ile = 0.001, Leu = 0.002, Leu = 0.002, Tyr = 0.002, Phe = 0.008, His = 0.003, Lys = 0.000, and Arg = 0.000.

**Table 6:** Macro-mineral content (mg/100 g on an as-is basis) of royal jelly samples (superscripts indicates significant differences, *p* < 0.05; lower case superscript indicates the significant difference among the RJ samples and upper case superscript indicates significant difference between treatment groups).

Feed Supplement	Sample No.	Calcium	Magnesium	Potassium	Sodium	Phosphorus	Iron	Zinc	Copper	Manganese
Sugar syrup	S1	12.7 ± 0.40 ^ab^	28.4 ± 0.90 ^a^	283.6 ± 13.25 ^ab^	1.9 ± 0.06 ^bcd^	212.0 ± 11.25 ^a^	1.1 ± 0.04 ^abcd^	2.4 ± 0.11 ^ab^	0.4 ± 0.02 ^a^	0.1 ± 0.01 ^ab^
	S2	15.0 ± 0.08 ^cd^	36.2 ± 0.65 ^bc^	334.1 ± 11.40 ^de^	1.7 ± 0.04 ^ab^	263.1 ± 4.05 ^bc^	1.1 ± 0.08 ^abcd^	2.7 ± 0.01 ^b^	0.6 ± 0.04 ^de^	0.1 ± 0.01 ^abc^
	S3	13.9 ± 0.24 ^bc^	33.4 ± 0.78 ^b^	303.7 ± 2.56 ^bc^	1.7 ± 0.07 ^a^	243.5 ± 5.25 ^b^	0.9 ± 0.05 ^a^	2.4 ± 0.09 ^ab^	0.5 ± 0.02 ^abc^	0.1 ± 0.00 ^abc^
	S4	11.5 ± 0.35 ^a^	27.0 ± 1.65 ^a^	261.9 ± 28.79 ^a^	1.7 ± 0.28 ^a^	204.9 ± 9.09 ^a^	1.0 ± 0.05 ^ab^	2.3 ± 0.15 ^ab^	0.4 ± 0.07 ^ab^	0.1 ± 0.01 ^abc^
	S5	12.3 ± 0.93 ^ab^	29.3 ± 2.03 ^a^	273.7 ± 18.21 ^a^	2.0 ± 0.25 ^cd^	209.8 ± 18.03 ^a^	1.0 ± 0.14 ^ab^	2.3 ± 0.34 ^a^	0.5 ± 0.07 ^bcd^	0.1 ± 0.01 ^a^
Honey	H1	13.5 ± 0.65 ^bc^	33.0 ± 0.89 ^b^	337.6 ± 8.69 ^de^	1.7 ± 0.01 ^a^	245.4 ± 3.28 ^b^	1.2 ± 0.09 ^d^	2.6 ± 0.11 ^ab^	0.6 ± 0.02 ^de^	0.2 ± 0.02 ^cd^
	H2	13.7 ± 0.60 ^bc^	33.3 ± 1.94 ^b^	313.4 ± 20.03 ^cd^	2.1 ± 0.07 ^d^	239.9 ± 16.68 ^b^	1.0 ± 0.12 ^abc^	2.4 ± 0.12 ^ab^	0.5 ± 0.06 ^cde^	0.1 ± 0.02 ^abcd^
	H3	14.9 ± 1.47 ^cd^	33.7 ± 3.81 ^b^	350.1 ± 14.13 ^e^	1.9 ± 0.08 ^abc^	245.8 ± 19.73 ^b^	1.2 ± 0.11 ^bcd^	2.6 ± 0.15 ^ab^	0.5 ± 0.08 ^de^	0.1 ± 0.04 ^abcd^
	H4	14.6 ± 1.36 ^cd^	34.4 ± 2.21 ^b^	335.5 ± 12.44 ^de^	1.8 ± 0.11 ^abc^	245.5 ± 17.99 ^b^	1.2 ± 0.15 ^cd^	2.5 ± 0.26 ^ab^	0.5 ± 0.04 ^cde^	0.2 ± 0.10 ^d^
	H5	15.5 ± 0.45 ^d^	38.4 ± 1.01 ^c^	362.0 ± 12.82 ^e^	2.6 ± 0.16 ^e^	280.1 ± 5.85 ^c^	1.1 ± 0.05 ^abcd^	2.6 ± 0.13 ^b^	0.6 ± 0.04 ^e^	0.1 ± 0.01 ^abc^
	H6	13.7 ± 0.43 ^bc^	35.2 ± 0.97 ^bc^	338.1 ± 13.29 ^de^	2.1 ± 0.18 ^cd^	254.6 ± 10.53 ^b^	1.1 ± 0.15 ^bcd^	2.4 ± 0.11 ^ab^	0.6 ± 0.03 ^de^	0.2 ± 0.03 ^bcd^
Mean (sugar syrup)		13.1 ± 1.38	31.5 ± 3.82	291.4 ± 28.37 ^B^	1.8 ± 0.14	226.7 ± 25.42	1.0 ± 0.08	2.4 ± 0.16	0.5 ± 0.08	0.1 ± 0.00
Mean (honey)		14.3 ± 0.81	34.7 ± 1.99	339.5 ± 16.26 ^A^	2.0 ± 0.32	251.9 ± 14.61	1.1 ± 0.08	2.5 ± 0.10	0.6 ± 0.05	0.2 ± 0.05
*p* *		0.127	0.090	0.015	0.152	0.097	0.050	0.292	0.152	0.076

* *p* value (two-tail) of the *t*-test, assuming unequal variance conducted between the two treatment groups.

## Data Availability

The original contributions presented in the study are included in the article, further inquiries can be directed to the corresponding author.
